# Preliminary characterizations of a serum biomarker for sarcoidosis by comparative proteomic approach with tandem-mass spectrometry in ethnic Han Chinese patients

**DOI:** 10.1186/1465-9921-14-18

**Published:** 2013-02-11

**Authors:** Yuan Zhang, Xianqiu Chen, Yang Hu, Shanshan Du, Li Shen, Yifan He, Yuxuan Zhang, Xia Zhang, Huiping Li, Rex C Yung

**Affiliations:** 1 Department of Respiratory Medicine, Shanghai Pulmonary Hospital, Tongji University School of Medicine, 200433, Shanghai, China; 2 Department of Pulmonary and Critical Care Medicine, Johns Hopkins University School of Medicine, 21205, Baltimore, MD, USA

**Keywords:** Sarcoidosis, Diagnosis, Proteomics, Biomarker, Serum Amyloid A

## Abstract

**Background:**

The diagnosis of sarcoidosis is still a significant challenge in China because of the need to exclude other diseases including granulomatous infections and malignancies that may be clinically and radiographically similar. The specific aim of the study is to search for serum protein biomarkers of sarcoidosis and to validate their clinical usefulness in differential diagnosis.

**Methods:**

Serum samples were collected from patients with sarcoidosis (n = 37), and compared to those from patients with tuberculosis (n = 20), other pulmonary diseases (n = 20), and healthy volunteers (n = 20) for determination of sarcoidosis-specific or -associated protein expression profiles. The first part of this study focused on proteomic analysis of serum from patients with sarcoidosis to identify a pattern of peptides capable of differentiating the studied populations using the ClinProt profiling technology based on mass spectrometry. Enzyme Linked Immunosorbent Assay (ELISA) was then used to verify corresponding elevation of the serum protein concentration of the potential biomarkers in the same patients sets. Receiver operating characteristic curve (ROC) analyses was performed to determine the optimal cutoff value for diagnosis. Immunohistochemistry was carried out to further confirm the protein expression patterns of the biomarkers in lung tissue.

**Results:**

An unique protein peak of M/Z 3,210 Daltons (Da) was found to be differentially expressed between the sarcoidosis and control groups and was identified as the N-terminal peptide of 29 amino acids (94-122) of serum amyloid A (SAA). ELISA confirmed that the serum SAA level was significantly higher in the sarcoidosis group than that of the other 3 control groups (*p* < 0.05). The cutoff for serum SAA concentration determined by ROC analysis was 101.98 ng/ml, with the sensitivity and specificity of 96.3% and 52.5%, respectively. Immunohistochemical staining showed that the SAA depositions in lung tissue of the sarcoidosis patients were also significantly more intense than in non-sarcoid lung tissue (*p* < 0.05).

**Conclusion:**

This is the first study to investigate serum protein markers in Chinese subjects with sarcoidosis. This study shows that the serum SAA expression profiles were different between the sarcoidosis and non-sarcoidosis groups. SAA may be a potential serum biomarker for ruling-out the diagnosis of sarcoidosis in Chinese subjects.

## Background

Sarcoidosis is a systemic disease of unknown etiology, with the pathological feature of non-caseous epithelioid granulomas. Many organs could be involved, and especially the lung and intrathoracic lymph nodes [1,2]. Sarcoidosis is a diagnosis of exclusion because of its similarity in the clinical presentations to other lung diseases (such as tuberculosis, fungal infections, lung cancer, and cryptogenic organizing pneumonia) [3]. The confirmatory diagnosis of sarcoidosis is established only when clinicoradiographic findings are supported by histological evidence of non-caseating granulomatous inflammation and other causes of granulomas and local reactions have been reasonably excluded [2,3]. China has a high prevalence of TB, which results in a significant challenge in differentiating sarcoidosis from other granulomatous lung diseases especially in cases of smear negative tuberculosis [4,5]. Thereby highly sensitive and selective serum biomarker assay will be useful for the diagnosis of sarcoidosis.

Although great efforts have been devoted to establish methodologies that can differentiate sarcoidosis from other lung diseases [4-7], the diagnosis of sarcoidosis still requires histological confirmation of the presence of granulomatous inflammation and at the same time to exclude other potential causes of granulomatous inflammation. However, tissue biopsy is cost-prohibitive and invasive, and the patients with sarcoidosis presenting with pulmonary findings that are suggestive of sarcoidosis often are not diagnosed until much later, resulting in delay in treatment, and this may be especially detrimental if the final diagnosis turns out to be an infectious process (TB) or cancer. Therefore, a less invasive, less expensive, sensitive and specific test with a rapid turn-around time would be clinically useful for the early diagnosis of sarcoidosis. Serum markers that can be measured conveniently, such as serum amyloid A (SAA), soluble interleukin-2 receptor (sIL-2R), lysozyme, angiotensin-converting enzyme (ACE), and the glycoprotein KL-6, have been reported [6-8]. However, no single biomarker is sufficiently sensitive and specific to be recommended for clinical use [8].

Differential proteomics is the systematic investigation of changes in patterns of serum proteins in diseases [9]. New technologies used in proteomics research such as the ClinProt profiling technology (a Matrix-Assisted Laser Desorption/ Ionization Time of Flight Mass Spectrometry (MALDI-TOF-MS) combined with magnetic beads for capture of analyte), have been widely used in the search for novel diagnostic biomarkers and as therapeutic targets. Biomass spectrometry is featured with high-throughput, high-sensitivity and high-resolution, and therefore has been used for exploring the differential protein expression patterns in serum. Substantial progresses have been made in cancer and autoimmune diseases diagnosis using the technology of mass spectrometry [10,11].

In the first part of this study, we used ClinProt profiling technology to discovery serum protein biomarkers associated with sarcoidosis. Serum amyloid A (SAA) was increased in the serum of patients with sarcoidosis, compared with non-sarcoidosis groups including healthy controls.

Serum amyloid A (SAA) is a protein that is secreted by the liver during an acute phase of http://inflammation[12-20]. The association of SAA and sarcoidosis has been previously reported [13-15,18,19]. In 1989, Rubinstein et. al. measured SAA concentrations in 25 sarcoidosis patients and 94 healthy volunteers. This study showed that SAA concentrations increased significantly in the sarcoidosis group as compared to the healthy volunteers [13]. An Italian study also found that serum concentrations of SAA were significantly higher in sarcoidosis patients than in healthy controls [15]. However, previous related studies had only enrolled healthy volunteers as control. In addition to sarcoidosis, SAA is also associated with other diseases such as tuberculosis, chronic obstructive pulmonary disease (COPD) and lung cancer, all of which may have elements of inflammatory reaction in their pathophysiology [16-18]. Recently, Chen ES and colleagues proposed SAA as a key regulator of granulomatous inflammation in Sarcoidosis [14], hence, any new study should also include non-sarcoid inflammatory lung diseases as comparator groups. As existing studies have originated from North America and from Europe, SAA have also not been investigated in a cohort of Chinese saroidosis patients.

On the second part of the study we evaluated the effectiveness of SAA as a diagnostic biomarker of Chinese sarcoidosis compared with non-sarcoidosis groups that include healthy controls and patients with tuberculosis, interstitial lung diseases and lung cancer.

## Patients and methods

### Patients and diagnostic criteria

Patients with tissue proven sarcoidosis, tuberculosis (TB), or other lung diseases (including chronic obstructive pulmonary disease (COPD), interstitial lung disease and lung cancer) who were evaluated at the Shanghai Pulmonary Hospital between September 2008 and April 2010 were recruited into this study. 26 healthy volunteers were also enrolled as normal controls. Demographics of the study subjects are presented in Table 1.

**Table 1:** Demographic information of subjects recruited for serum studies

**Category**	**Sarcoidosis group**	**Control subjects**		
		**Healthy group**	**Tuberculosis group**	**Other pulmonary disease group**
**Total subjects, n**	64^*^	26	35	38^▲^
**Age, years**	48	49	45	46
**Gender**				
**Male**	24	10	13	14
**Female**	40	16	22	24
**CXR stage**	0/18/38/8/0	N/A	N/A	N/A
**0/I/II/III/IV**^**§**^				

^§^ CXR = chest X-ray; N/A = not applicable; CXR stage: 0 = no adenopathy, no lung infiltrates; stage I = hilar & mediastinal adenopathy only; stage II = hilar & mediastinal adenopathy plus lung infiltrates; stage III = lung infiltrates only; stage IV = pulmonary fibrosis.

* All of the 64 case, 44 were not being treated, 20 were treated with corticosteroid, CXR stage as stated.

^▲^ Other Pulmonary Disease Group including 10 cases of interstitial lung disease, 7 cases of chronic obstructive pulmonary disease (COPD), and 11 cases of lung cancer.

The proposed study was approved by Biomedical Ethics Review (No. 2011-FK-10) of the Department of Medicine and Life Sciences, Tongji University School of Medicine. Informed consent was obtained from each patient who underwent blood draw for serum analysis and tissue biopsy prior to inclusion in this study.

### Sample collection and processing

Fasting specimen of 5 ml of peripheral venous blood was drawn from each subject, and placed at room temperature for 30-60 minutes. After centrifugation at 3,000 rpm for 15 minutes, the serum was dispensed into as an aliquot of 0.5 ml/vial for storage at -80°C till they were assayed.

### MS analysis and statistical processing

For the ClinProt system proteomics analysis, serum samples were collected from patients with sarcoidosis (n = 37), patients with tuberculosis (n = 20), patients with other pulmonary diseases (n = 20), and healthy volunteers (n = 20) for determination of sarcoidosis-specific or -associated protein expression profiles. Before ClinProt system (Bruker Daltonics Inc., USA) analysis, the serum samples were pre-treated with Profiling Kit 100 MB-WCX following the manufacturer's protocol. The limit of molecular weight (Mr) error of the ClinProt system for data acquisition was set at <0.1% prior to the procedure. Angiotensin-II (containing 11 peptides with average Mr bias <0.01%) was used as an external standard, and the system was re-calibrated after every 8 acquisitions. Thirteen standard sera were used for quality controls, and the resultant testing peak had a coefficient of variation of < 30% indicative of acceptable protocol stability. The instrumental parameters were: a) positive ion spectrometry b) ion source accelerating voltage of 20 kV, c) ion extraction delay of 120 ns, d) the laser frequency of 25 Hz, e) the highest detected protein Mr of 20,000, and f) the optimized range of M/Z 800-10,000 and average 400 point signals for each sample. Flex Analysis 3.0 software was used for data acquisition and pre-processing to obtain the average serum protein profiles of each group, followed by multiplexed comparisons.

**Figure 1:**
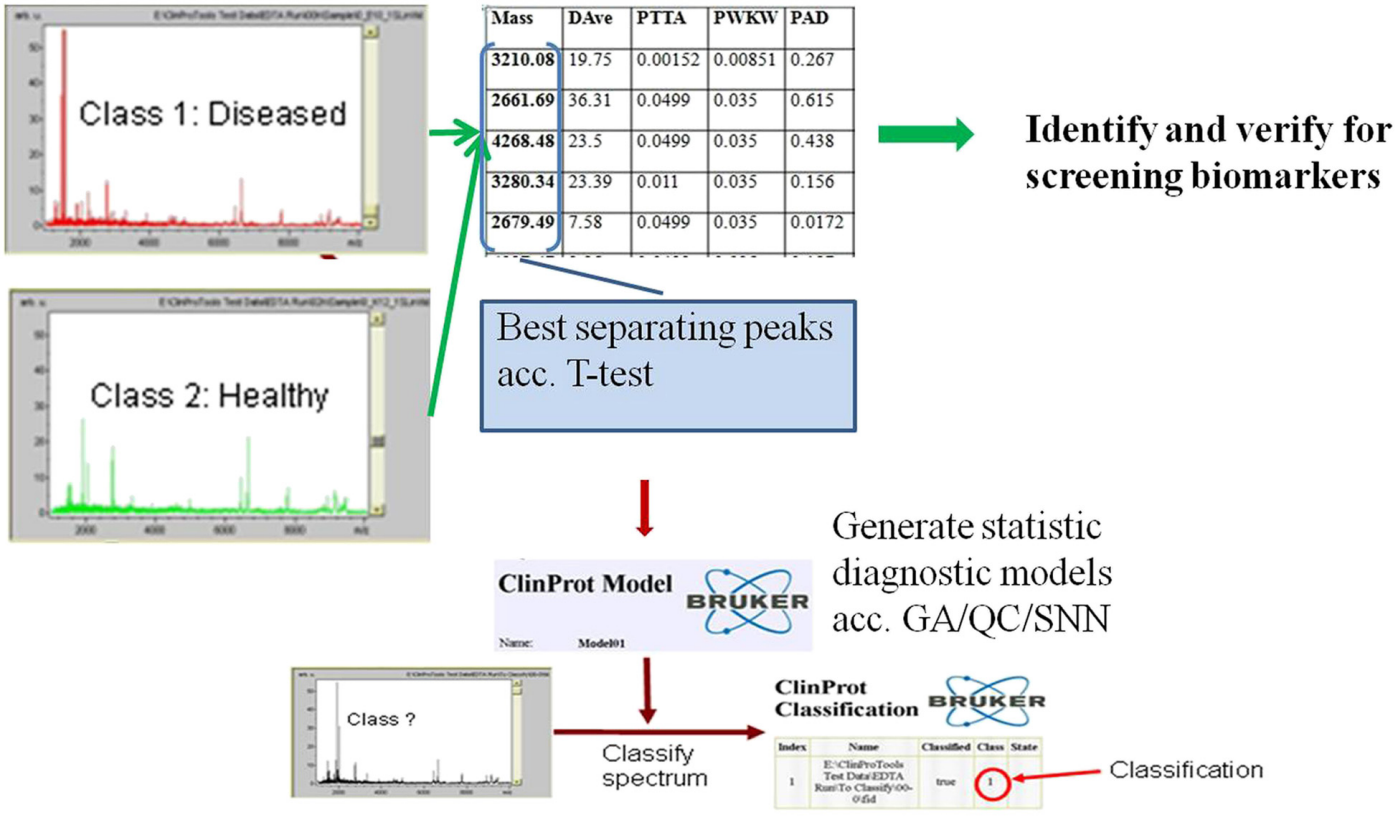
The acquired data of the protein peaks were statistically analyzed.

The acquired data of the protein peaks were analyzed by the Anderson-Darling test, pairwise comparison t-test, and the Wilcoxon test to determine whether differences exist in candidate protein biomarkers levels between sarcoidosis and non-sarcoidosis groups. In addition, the ClinProt system including analytical software bundle, the ClinProTools that comprise of three machine-learning algorithms (Genetic Algorithm (GA), Supervised Neural Network (SNN) and QuickClassifier (QC)) for generating classification patterns of the serum protein distributions. This package of machine-learning algorithms is able to analyze serum protein profile of sarcoidosis and non-sarcoidosis subjects as shown in Figure 1.

### Identification of differentially expressed protein peaks by LC-MS/MS

The sequence of differential peptide peaks between sarcoidosis group and non-sarcoidosis groups was identified by LC-MS/MS (NanoAquity UPLC liquid chromatograph, Waters, USA; LTQ Orbitrap XL electrospray ionization mass spectrometer, Thermo Fisher Scientific). All MS/MS spectra were identified by using SEQUEST [v.28 (revision 12), Thermo Electron Corp.] against the human International Protein Index (IPI) database. The searching parameters were set up as follows: NoEnzyme, the variable modification was oxidation of methionine, the peptide mass tolerance was 5 ppm, and the fragment ions tolerance was 1 Da. Positive protein identification was accepted for a peptide with Xcorr of greater than or equal to 3.00 for triply and 2.50 for doubly charged ions, and all with ΔCn ≥ 0.1, peptide probability ≤ 0.001.

### Detection of Serum Amyloid A (SAA) in serum

To follow up the finding by mass spectrometry, human SAA ELISA assay (Y10903A, Human Serum amyloid A, SAA ELISA KIT) was used to measure the SAA level in 64 serum samples from patients with sarcoidosis. 99 serum samples were obtained from the comparison groups (26 healthy volunteers, 35 tuberculosis cases, 12 COPD cases, 14 interstitial lung disease cases and 12 lung cancer cases). Twenty (20) of 64 sarcoidosis patients were classified as the “treated” group, who had been successfully treated by corticosteroid and are showing improvement to complete cure. The remaining 44 patients were determined as “untreated” sarcoidosis, who had clinical symptoms to different extents but had not receive corticosteroid.

### Preliminary evaluation of the clinical performance characteristics of the SAA

The receiver operating characteristic (ROC) curve was plotted according to the SAA contents, as determined by the ELISA with the values of SAA in the sarcoidosis and non-sarcoidosis subjects. The optimal cut-off threshold is determined by ROC as the maximal value of Youden index (Youden index = sensitivity + specificity-1) for the quantitative analysis.

### Detection of SAA in lung tissue

The streptavidin-perosidase (SP) immunohistochemical assay was performed to detect the expression of SAA by a SAA antibody (clone mc1; Dako; Carpinteria, CA, USA) in tissue biopsies of a subset of our test subjects: 10 patients with sarcoidosis (lung in 2, subcutaneous granulomas in 3, and lymph nodes in 5) and lung tissues of 10 patients with other lung diseases (tuberculosis in 2, organizing pneumonia in 2, cryptococcal pneumonia in 2, lung carcinoma in 4).

### Statistics

All experiments were carried out in triplicates, and the data were presented as mean ± standard deviation (SD). The statistical software SPSS 11.0 was used for data analysis. Independent-samples t test assuming equal variance and ANOVA were performed with p < 0.05 indicated statistically significant difference.

## Results

### Reproducibility of the ClinProt system

**Figure 2:**
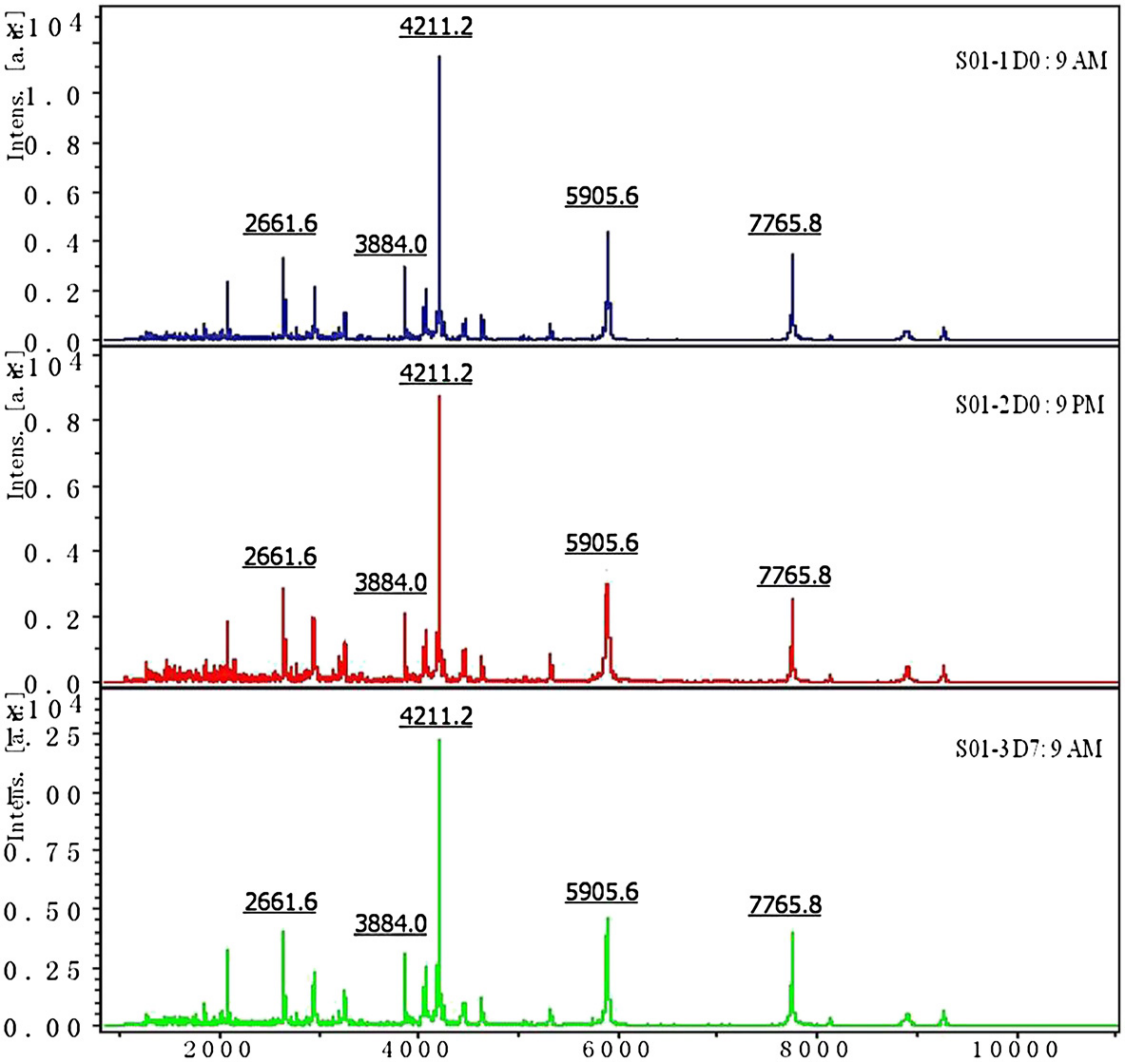
**The triplicate serum mass spectra of a representative sample at various time points under the standard conditions.** X-axis represents mass to charge ratio, and Y-axis represents the intensity (expression levels).

Two samples from patients with sarcoidosis and one sample from a healthy control were used to verify reproducibility of the ClinProt system. Proteins were detected in these samples in a parallel experiment. The results showed that under the above-mentioned experimental conditions, M/Z bias was in a statistically acceptable range of errors (coefficient of variation or CV < 30%). Figure 2 shows the triplicate mass spectra of a representative serum sample at various time points. The overall profiles of the 67 protein peaks were comparable. The average CV was 13.5%.

### Comparison of the mean serum protein profiles

In the range of M/Z 800-10,000, there were 8, 8 and 13 different protein peaks when the sarcoidosis subjects were compared with healthy controls, tuberculosis subjects and other lung disease subjects respectively. The protein peaks were then analyzed using the ClinProTools machine learning algorithms (GA, QC and SNN) to determine whether the identification and predictive ability of classification models in distinguishing sarcoidosis from the other disease subjects and from healthy controls (Table 2).

**Table 2:** Differential distribution of peaks identified by the different classification algorithms of the Clinprot system

	**Differential peaks (M/Z)**	**Diagnostic models**		
		**GA** **Identifica-tion and predictive ability**	**QC** **Identifica-tion and predictive ability**	**SNN** **Identifica-tion and predictive ability**
**Sarcoidosis vs.**	***3210***, 2661, 3280, 2679↑;	91.35%;	85.09%;	93.15%;
**healthy control**	4268, 4397, 6433, 4211↓;	66.76%	68%	69.42%
**Sarcoidosis vs.**	3976, 3955, ***3210***, 3159, 3280↑;	97.22%;	88.89%;	100%;
**tuberculosis**	2901, 2989, 2661↓;	75.11%	67.38%	78.62%
**Sarcoidosis vs.**	2954, 2106, 4469, ***3210****,*1279, 6631, 2933↑;	95.63%;	88.24%;	100%;
**other lung diseases control**	5266, 5808, 5292, 5337, 5484. 5249↓.	82.02%	80.57%	81.62%

Specifically, of the total 8 differential peaks between the sarcoidosis group and tuberculosis group, 5 protein peaks (M/Z 3,976.97; 3,955.19; 3,210.04; 3,159.37 and 3,280.43) were overexpressed in sarcoidosis versus the other 3 (M/Z 2,901.48; 2,989.71 and 2,661.63) that were more elevated and overexpressed in the tuberculosis group. In our study, the SNN method achieved the best results in identification and predictive capability.

**Figure 3:**
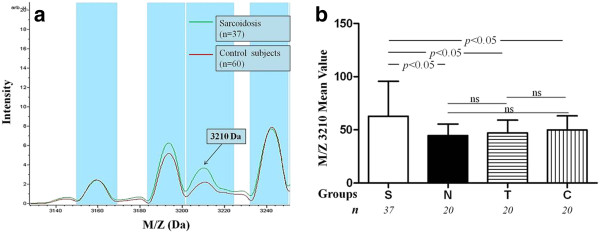
**The expression level of the protein peak M/Z 3,210.** The mean intensity of different expressed proteins peak M/Z 3,210 in the sarcoidosis group was significantly higher when compared to non-sarcoidosis groups (S: sarcoidosis group, N: healthy control, T: tuberculosis group, C: diseased group).

In addition, as shown in Figure 3, the expression level of the protein peak M/Z 3,210 in the sarcoidosis group was significantly higher than that of the non-sarcoidosis groups (*p* < 0.05), but showed no significant differences between the non-sarcoidosis groups (healthy control, tuberculosis, and other diseased subjects, *p* > 0.05), which indicated that this is a disease-associated or specific protein for saroidosis.

### *M/*Z *3,210 was identified as Serum amyloid A protein*

The LC-MS/MS trapped a molecular species of M/Z 3,211.57 (M + H), and the tandem MS analysis in the second stage obtained the parent ion of +4 charged 804.15. After LC-MS/MS data acquisition, the SEQUEST of IPI Human (3.45) database retrieved the consistent peptide identity of "SLADQAANEWGRSGKDPNHFRPAGLPEKY", which is the N-terminal 29 peptide of SAA (94-122; SAA, IPI:IPI00552578.2 Gene_Symbol = SAA1; SAA2 Serum amyloid A protein precursor) (Table 3).

**Table 3:** The listed differential peaks to be identified

**Scan(s)**	**MH+**	**Peptide**	**Reference**	**P (pep)**
1681	3211.57	SLADQAANEWGRSGKDPNHFRPAGLPEKY	IPI:IPI00552578.2 Gene_symbol = SAA1;SAA2 Serum amyloid a protein precursor	1.56E-04

Scan: scan number; MH+: the mass to charge ratio by adding H+; Reference: protein code and name; Peptide: peptide sequence; P (pep): degree of confidence.

### Verification of SAA in the serum

**Figure 4:**
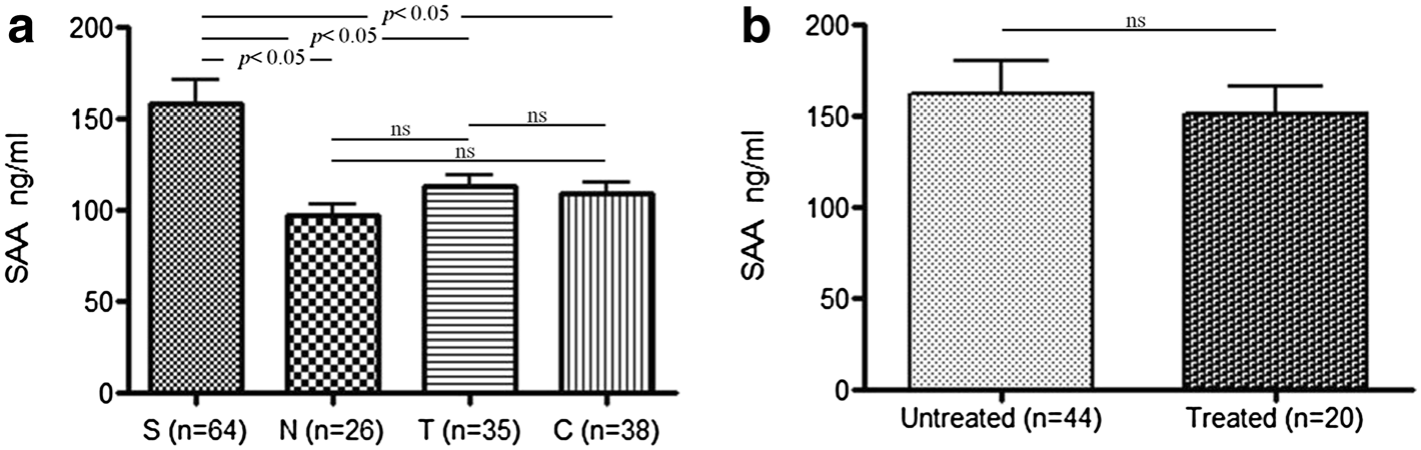
**The SAA levels were significantly elevated in sarcoidosis group, detected by ELISA in serum.** (**a**) The SAA levels of the sarcoidosis group were significantly higher (p <0.05) than those of non-sarcoidosis groups. S: sarcoidosis group, N: healthy control, T: tuberculosis group, C: diseased control group). (**b**) There was no significiant difference between treated and untreated groups.

Having the identified M/Z 3,210 as the N-terminal peptide of SAA, next ELISA was employed to evaluate the expression of SAA in serum of sarcoidosis and non-sarcoidosis subjects. As shown in Figure 4, the levels of SAA in serum of the sarcoidosis group (both treated and untreated groups) were significantly elevated than those of the non-sarcoidosis group (p < 0.05). ELISA was performed to detect SAA in serum from the patients with sarcoidosis and the higher levels of SAA were more likely to be associated with sarcoidosis than with non-sarcoidosis.

### Evaluation of the usefulness of serum SAA levels for clinical diagnosis of sarcoidosis by ROC

**Figure 5:**
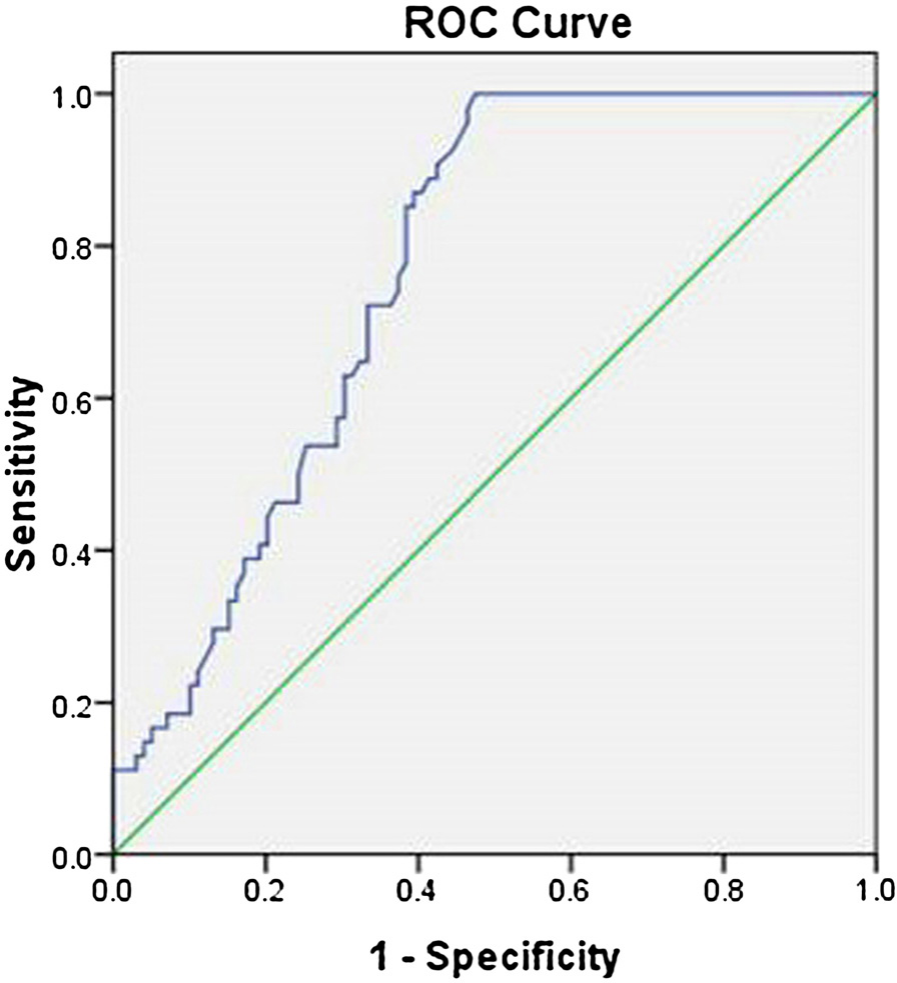
The cutoff of SAA contents in serum detected by semi-quantitative ELISA is used to determine sarcoidosis (n = 64) and non-sarcoidosis (n = 99) of the receiver operating characteristic (ROC) curve.

SAA in serum of 163 subjects (64 sarcoid and 99 non-sarcoid) detected by semi- quantitative ELISA is plotted to generate the ROC curve using the cutoff point of 101.98 μg/L. The optimal cut-off yields the best sensitivity of 96.3%, and specificity of 52.5%, respectively; and the AUC of 0.763 (Figure 5).

### Verification of SAA in the lung tissue of patients with sarcoidosis

**Figure 6:**
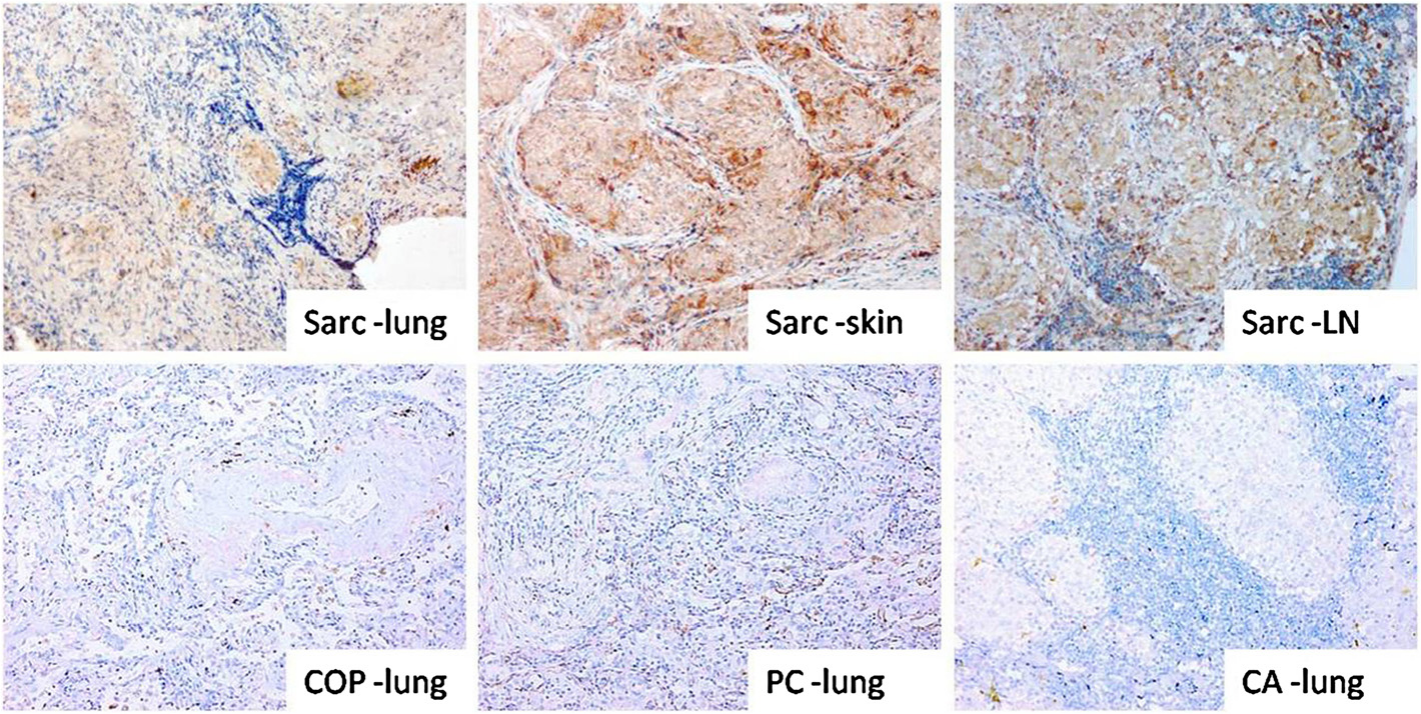
Immunohistochemistry showed SAA deposition in lung, subcutaneous and lymph node granulomas of patients with sarcoidosis, apparently thicker than the appearances in the lungs of patients with organized pneumonia, cryptococcal lung and lung carcinoma.

Immunohistochemical assay showed that SAA depositions occurred in lung, subcutaneous and lymph node granulomas of patients with sarcoidosis, and appeared more intense in tissue staining than in the lungs of patients with organized pneumonia, cryptococcal lung and lung cancer, respectively (Figure 6).

## Discussion

The diagnosis of sarcoidosis is still a significant challenge in China because of the need to exclude lung diseases that are especially common in China (ie. TB, COPD, lung cancer of which in patients with similar clinical and/or radiographic findings). This is the first study demonstrating potential effectiveness of protein profiling in serum with ClinProt as a diagnostic tool for sarcoidosis in ethnic Han Chinese. We confirm that the SAA serum level and tissue expression of sarcoidosis are higher than in other lung diseases.

Over the last decade, accumulated evidences in the research of protein biomarkers of sarcoidosis have shown that it is possible to find sarcoidosis-specific or associated proteins through analysis of body fluids. Researchers used to compare protein profiles from sarcoidosis patients with healthy controls in specimens of bronchoalveolar lavage fluid (BALF) [21-26]. However, no further analysis for the positive identification of the sarcoidosis-associated peak was performed. Collection of blood is much less invasive than collection of BALF, and not subject to variabilities of BALF collection such as dilution and contamination by oropharyngeal secretions. Several studies have reported novel serum biomarkers in sarcoidosis. Bons JA and his colleagues from The Netherlands first applied surface-enhanced laser desorption ioni-zation time-of-flight mass spectrometry (SELDI-TOF-MS) to compare protein profiles from 35 sarcoidosis patients to 35 healthy controls in 2007 [27]. The differential protein peak M/Z 17,377 was identified as the alpha-2 chain of haptoglobin, although the total haptoglobin level in the serum detected by ELISA was not statistically significant between the disease group and the controls. In the same study, there were two other unidentified serum proteins that were also up-regulated in sarcoidosis patients. Recently, Bargagli et al, using proteomic tools found that serum concentrations of SAA were significantly higher in sarcoidosis patients than in healthy controls. They thought that SAA is likely one of the unidentified biomarkers in Bons’s proteomic study [15,27].

This study is the first effort using the ClinProt system of serum protein profiling in Chinese subjects to discover new diagnostic biomarkers in sarcoidosis. The ClinProt system is considered as more advanced technologies than SELDI-TOF-MS [28]. We are charged with the need to differentiate sarcoidosis from other similar diseases with enrolling 37 patients with sarcoidosis, 20 healthy volunteers and otherwise non-sardoidosis diseases in lung including tuberculosis, COPD, interstitial lung disease and lung cancer as control. In our pilot study, among the multiple differential protein peaks detected by ClinProt-MALDI-TOF, the expression levels of M/Z 3,210 in the sarcoidosis group was significantly elevated in peak height than those in non-sarcoidosis groups (p < 0.05. Thus M/Z 3,210 was the potential candidate biomarker, which was further analyzed by LC-MS/MS. The investigations confirmed a 29 amino acid peptide sequence of “SLADQAANEWGRSGKDPNHFRPAGLPEKY”, which corresponds to the N-terminal fragment of SAA.

SAA is an acute phase protein, which belongs to a group of heterogeneous proteins of the apolipoprotein family. It is possible that SAA could activate the NF-kB signaling pathway through interactions with Toll-like receptor 2 (TLR2), leading to the development of chronic granuloma as has been recently reported by Chen et al [14]. Although the underlying mechanism of the increase in the secretion of SAA or the chemotatic tissue deposition due to changes in the internal environment remain unclear. Nonetheless these findings suggest that SAA could be a potential biomarker for the diseases.

A literature survey indicates that besides sarcoidosis, SAA is also over-expressed in several diseases such as COPD [16], lung cancer [17], and other interstitial lung diseases [18]. Our study showed that expression of SAA was significantly higher in sarcoidosis than in the other non-sarcoidosis disease groups (Figure 4). Furthermore, we found that sarcoidosis patients also had increased tissue levels of SAA, and it was deposited in the granulomas of patients (Figure 6).

The optimal cutoff of the serum SAA concentration obtained by the ROC analysis was 101.98 ng/ml yielding a sensitivity of 96.3% and specificity of 52.5% for diagnosis, respectively. By including not only healthy controls but by expanding the comparator groups to include patients with other inflammatory lung disease, our results demonstrated that SAA might be an important player in the development of sarcoidosis. However, although SAA is sensitive, it was less specific for the diseases tested in the study. This means that it is not for confirmative or for ruling- in a diagnosis of sarcoidosis.

A follow up question is whether the SAA is a marker of disease activity or it was a sequel of the treatments? As early as 1989, Rubinstein et al had shown that SAA concentration in 25 sarcoidosis patients (13 cases with “active sarcoidosis” and 12 cases with “inactive sarcoidosis”) and 94 healthy volunteers. He showed that SAA concentrations in both types of sarcoidosis were significantly higher than that of healthy controls. However, SAA was of limited usefulness as a marker for disease activity [13]. A report from Italy demonstrated that concentration of SAA in serum is significantly higher in patients with sub-acute onset requiring prolonged and multiple steroid treatments than in patients with sub acute onset but not requiring therapy (*p* < 0.001). In this study, we found that SAA is similarly increased in both “treated” and “untreated” sarcoidosis, which implies that SAA is not affected by the corticosteroid treatment.

Since the usefulness of multiple markers for diagnosis is now widely recognized [8]. ClinProt software could be used in combination with three different algorithms (Supervised Neural Network (SNN); Genetic Algorithm (GA) and QuickClassifier (QC) to create testable diagnostic models for classifying potential biomarkers with respect to whether they are associated with the sarcoidosis. In this study, the SNN method has achieved the optimal classification of sarcoidosis and non-sarcoidosis with efficiency of identification of 100%, and predictive capability of 78.62%; 93.15%, and 69.24% of efficiency of identification and predictive capability, respectively, between the sarcoidosis and the healthy controls; and 100% and 81.62% of efficiency of identification and predictive capability, respectively, between the sarcoidosis and the diseased comparator groups. The machine learning algorithms greatly improved the efficiency in identification of potential serum biomarkers for the diagnosis of sarcoidosis when compared to the previous study. GA mimics evolution in nature, together with the SNN and the QC are univariate sorting algorithms, which use the p-values at certain peak positions for classification. Therefore, these ClinProt system-based serological classification models can be applied to the clinical diagnosis of diseases, providing additional validation methods. However, the current classification models based on the ClinProt platform would be expensive and complicated to operate in routine clinical settings. Thus they are limited in wider clinical applications.

## Conclusion

In summary, our results provide evidences that the SAA expression profiles by ClinProt-MALDI-TOF technique were different between the sarcoidosis and controls in Chinese subjects. The finding that the elevation of the SAA N-terminal peptide is associated with sarcodosis, is confirmed by ELISA in serum and further verified by immunohistochemical staining in lung tissue. Moreover experimental data obtained with current study suggested that multiple markers for diagnosis could be used in differential diagnosis of sarcoidosis despite its limitation in of its specificity.

## Abbreviations

SAA: Serum amyloid A;Sarc: Sarcoidosis;TB: Tuberculosis;COPD: Chronic obstructive pulmonary disease;Da: Daltons;M/Z: The mass-to-charge ratio;ELISA: Enzyme Immunosorbent Assay;CV: Coefficient of variation;MALDI-TOF-MS: Matrix-Assisted Laser Desorption/ Ionization Time of Flight Mass Spectrometry;SELDI-TOF-MS: Surface-enhanced Laser Desorption/ Ionization Time of Flight Mass Spectrometry;SNN: Supervised Neural Network;GA: Genetic Algorithm;QC: Quick Classifier;ROC: Receiver operating characteristic curve

## Competing interests

The authors declare that they have no competing interests.

## Authors’ contributions

HL and YZ were responsible for experimental design and finalized the manuscript. XC and YH participated in the clinical sample collection and conducted the ClinProt study. SD participated in the data analysis and manuscript preparation. LS, YH, YZ, XZ were involved in data collection and clinical follow-up. RCY was responsible for editing and finalizing the manuscript. All authors read and approved the final manuscript.
